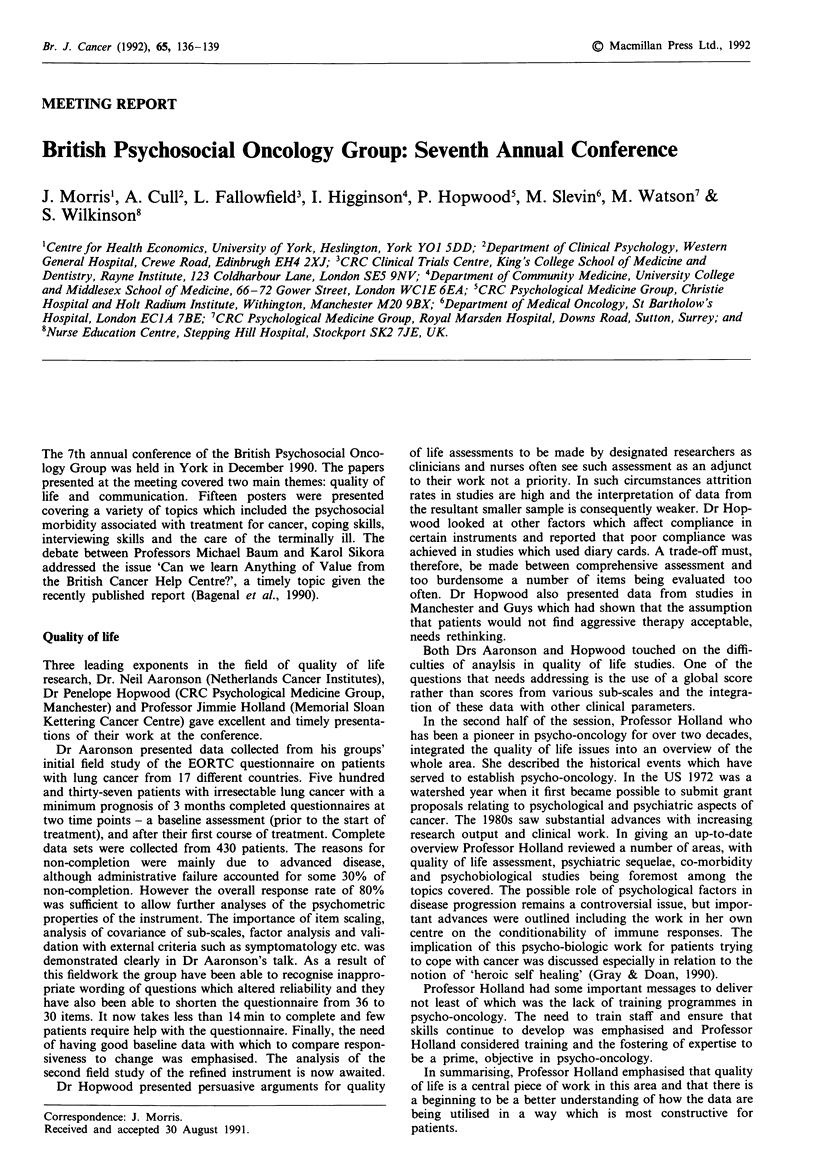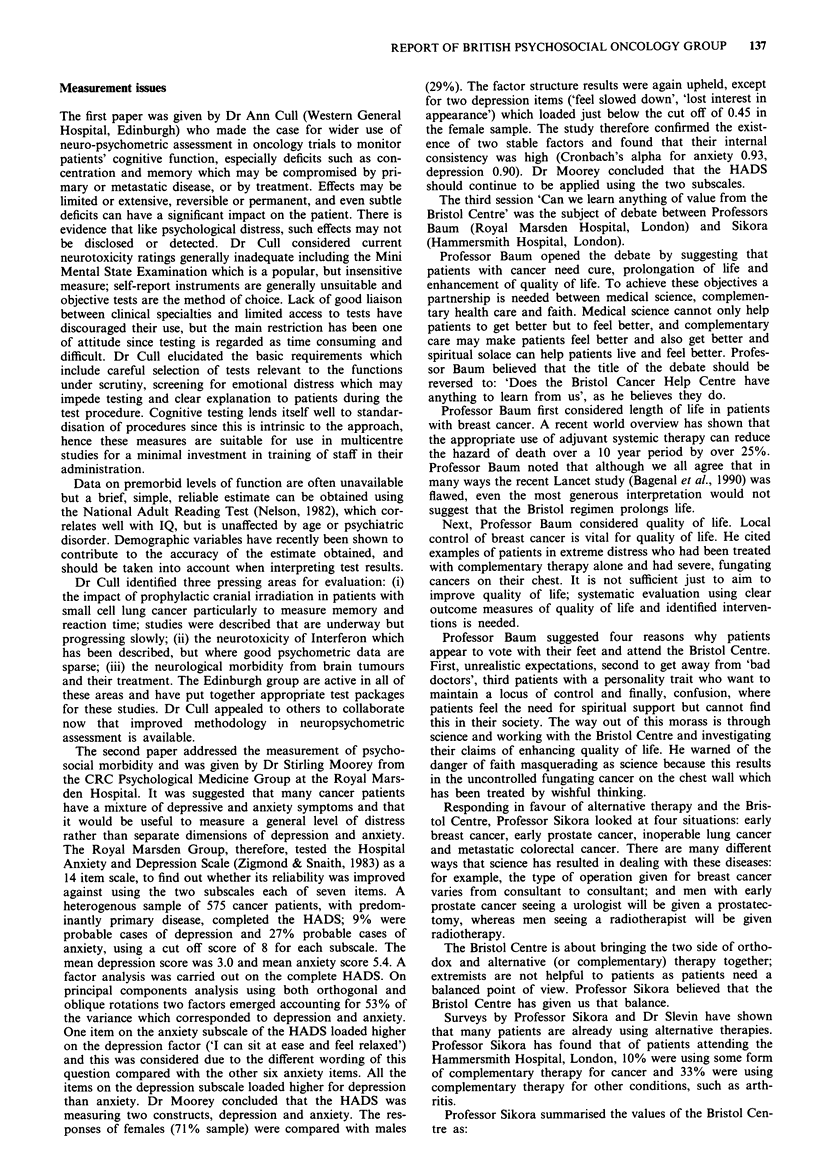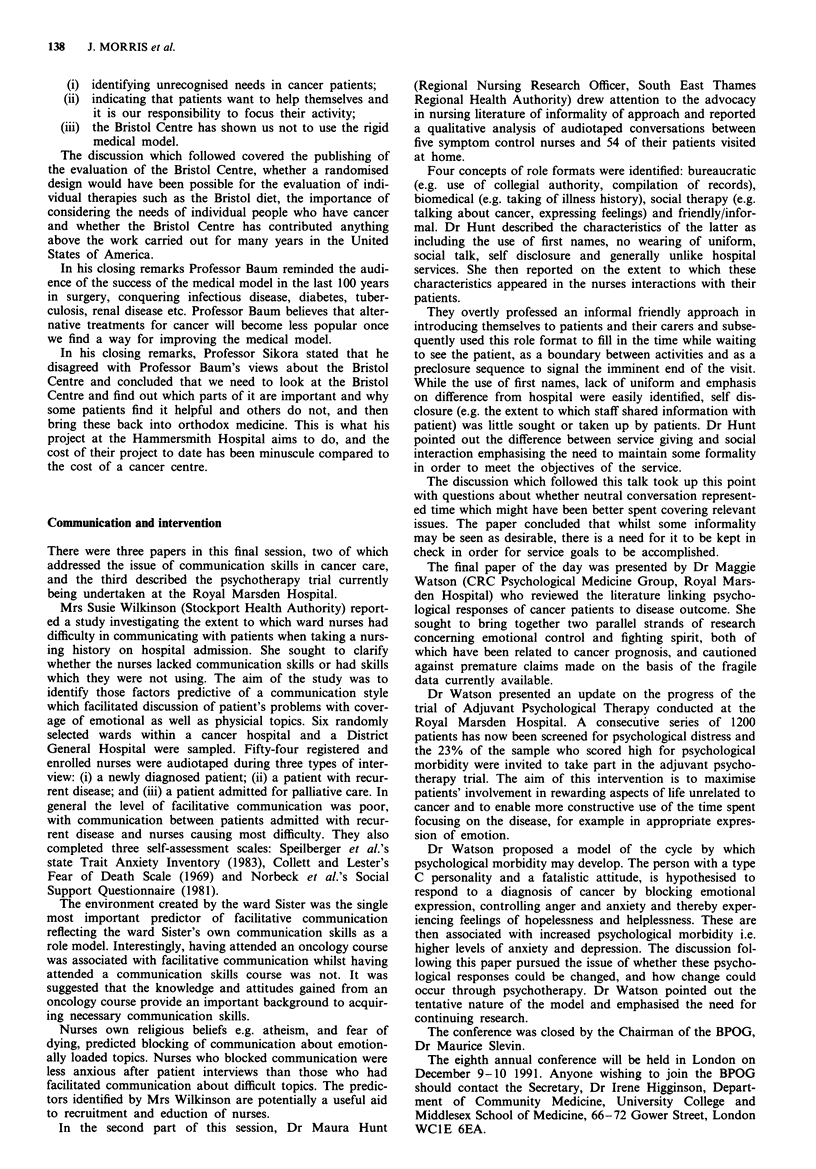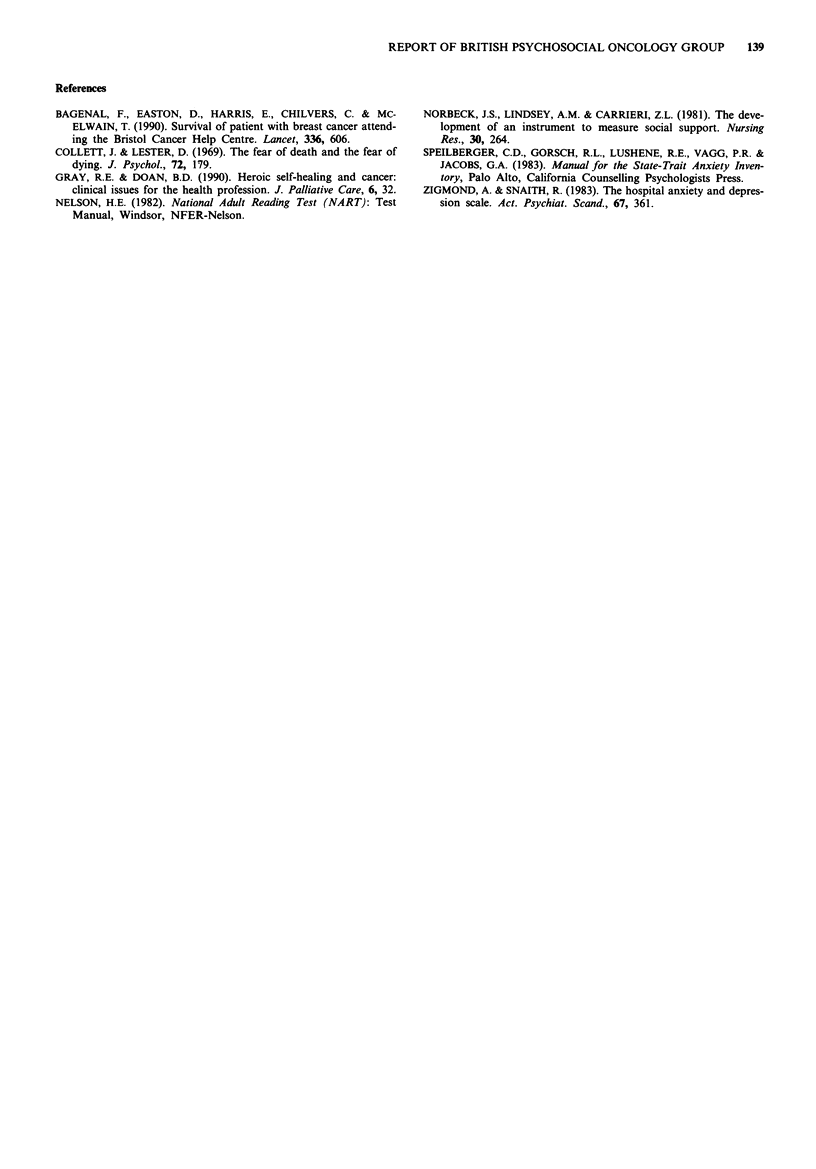# British Psychological Oncology Group: seventh annual conference.

**DOI:** 10.1038/bjc.1992.27

**Published:** 1992-01

**Authors:** J. Morris, A. Cull, L. Fallowfield, I. Higginson, P. Hopwood, M. Slevin, M. Watson, S. Wilkinson

**Affiliations:** Centre for Health Economics, University of York, Heslington, York Y01 5DD.


*Br. J. Cancer* (1992), **65**, 136–139 © Macmillan Press Ltd., 1992

MEETING REPORT

### British Psychosocial Oncology Group: Seventh Annual Conference

J. Morris[1], A. Cull[2], L. Fallowfield[3], I. Higginson[4], P. Hopwood[5], M. Slevin[6], M. Watson[7] & S. Wilkinson[8]

[1]*Centre for Health Economics, University of York, Heslington, York YO1 5DD;* [2]*Department of Clinical Psychology, Western General Hospital, Crewe Road, Edinbrugh EH4 2XJ;* [3]*CRC Clinical Trials Centre, King's College School of Medicine and Dentistry, Rayne Institute, 123 Coldharbour Lane, London SE5 9NV;* [4]*Department of Community Medicine, University College and Middlesex School of Medicine, 66–72 Gower Street, London WC1E 6EA;* [5]*CRC Psychological Medicine Group, Christie Hospital and Holt Radium Institute, Withington, Manchester M20 9BX;* [6]*Department of Medical Oncology, St Bartholow's Hospital, London EC1A 7BE;* [7]*CRC Psychological Medicine Group, Royal Marsden Hospital, Downs Road, Sutton, Surrey; and* [8]*Nurse Education Centre, Stepping Hill Hospital, Stockport SK2 7JE, UK.*

The 7th annual conference of the British Psychosocial Oncology Group was held in York in December 1990. The papers presented at the meeting covered two main themes: quality of life and communication. Fifteen posters were presented covering a variety of topics which included the psychosocial morbidity associated with treatment for cancer, coping skills, interviewing skills and the care of the terminally ill. The debate between Professors Michael Baum and Karol Sikora addressed the issue 'Can we learn Anything of Value from the British Cancer Help Centre?', a timely topic given the recently published report (Bagenal *et al.*, 1990).

### Quality of life

Three leading exponents in the field of quality of life research, Dr. Neil Aaronson (Netherlands Cancer Institutes), Dr Penelope Hopwood (CRC Psychological Medicine Group, Manchester) and Professor Jimmie Holland (Memorial Sloan Kettering Cancer Centre) gave excellent and timely presentations of their work at the conference.

Dr Aaronson presented data collected from his groups' initial field study of the EORTC questionnaire on patients with lung cancer from 17 different countries. Five hundred and thirty-seven patients with irresectable lung cancer with a minimum prognosis of 3 months completed questionnaires at two time points – a baseline assessment (prior to the start of treatment), and after their first course of treatment. Complete data sets were collected from 430 patients. The reasons for non-completion were mainly due to advanced disease, although administrative failure accounted for some 30% of non-completion. However the overall response rate of 80% was sufficient to allow further analyses of the psychometric properties of the instrument. The importance of item scaling, analysis of covariance of sub-scales, factor analysis and validation with external criteria such as symptomatology etc. was demonstrated clearly in Dr Aaronson's talk. As a result of this fieldwork the group have been able to recognise inappropriate wording of questions which altered reliability and they have also been able to shorten the questionnaire from 36 to 30 items. It now takes less than 14 min to complete and few patients require help with the questionnaire. Finally, the need of having good baseline data with which to compare responsiveness to change was emphasised. The analysis of the second field study of the refined instrument is now awaited.

Dr Hopwood presented persuasive arguments for quality of life assessments to be made by designated researchers as clinicians and nurses often see such assessment as an adjunct to their work not a priority. In such circumstances attrition rates in studies are high and the interpretation of data from the resultant smaller sample is consequently weaker. Dr Hopwood looked at other factors which affect compliance in certain instruments and reported that poor compliance was achieved in studies which used diary cards. A trade-off must, therefore, be made between comprehensive assessment and too burdensome a number of items being evaluated too often. Dr Hopwood also presented data from studies in Manchester and Guys which had shown that the assumption that patients would not find aggressive therapy acceptable, needs rethinking.

Both Drs Aaronson and Hopwood touched on the difficulties of anaylsis in quality of life studies. One of the questions that needs addressing is the use of a global score rather than scores from various sub-scales and the integration of these data with other clinical parameters.

In the second half of the session, Professor Holland who has been a pioneer in psycho-oncology for over two decades, integrated the quality of life issues into an overview of the whole area. She described the historical events which have served to establish psycho-oncology. In the US 1972 was a watershed year when it first became possible to submit grant proposals relating to psychological and psychiatric aspects of cancer. The 1980s saw substantial advances with increasing research output and clinical work. In giving an up-to-date overview Professor Holland reviewed a number of areas, with quality of life assessment, psychiatric sequelae, co-morbidity and psychobiological studies being foremost among the topics covered. The possible role of psychological factors in disease progression remains a controversial issue, but important advances were outlined including the work in her own centre on the conditionability of immune responses. The implication of this psycho-biologic work for patients trying to cope with cancer was discussed especially in relation to the notion of 'heroic self healing' (Gray & Doan, 1990).

Professor Holland had some important messages to deliver not least of which was the lack of training programmes in psycho-oncology. The need to train staff and ensure that skills continue to develop was emphasised and Professor Holland considered training and the fostering of expertise to be a prime, objective in psycho-oncology.

In summarising, Professor Holland emphasised that quality of life is a central piece of work in this area and that there is a beginning to be a better understanding of how the data are being utilised in a way which is most constructive for patients.

Correspondence: J. Morris.
Received and accepted 30 August 1991.

**Measurement issues**

The first paper was given by Dr Ann Cull (Western General Hospital, Edinburgh) who made the case for wider use of neuro-psychometric assessment in oncology trials to monitor patients' cognitive function, especially deficits such as concentration and memory which may be compromised by primary or metastatic disease, or by treatment. Effects may be limited or extensive, reversible or permanent, and even subtle deficits can have a significant impact on the patient. There is evidence that like psychological distress, such effects may not be disclosed or detected. Dr Cull considered current neurotoxicity ratings generally inadequate including the Mini Mental State Examination which is a popular, but insensitive measure; self-report instruments are generally unsuitable and objective tests are the method of choice. Lack of good liaison between clinical specialties and limited access to tests have discouraged their use, but the main restriction has been one of attitude since testing is regarded as time consuming and difficult. Dr Cull elucidated the basic requirements which include careful selection of tests relevant to the functions under scrutiny, screening for emotional distress which may impede testing and clear explanation to patients during the test procedure. Cognitive testing lends itself well to standardisation of procedures since this is intrinsic to the approach, hence these measures are suitable for use in multicentre studies for a minimal investment in training of staff in their administration.

Data on premorbid levels of function are often unavailable but a brief, simple, reliable estimate can be obtained using the National Adult Reading Test (Nelson, 1982), which correlates well with IQ, but is unaffected by age or psychiatric disorder. Demographic variables have recently been shown to contribute to the accuracy of the estimate obtained, and should be taken into account when interpreting test results.

Dr Cull identified three pressing areas for evaluation: (i) the impact of prophylactic cranial irradiation in patients with small cell lung cancer particularly to measure memory and reaction time; studies were described that are underway but progressing slowly; (ii) the neurotoxicity of Interferon which has been described, but where good psychometric data are sparse; (iii) the neurological morbidity from brain tumours and their treatment. The Edinburgh group are active in all of these areas and have put together appropriate test packages for these studies. Dr Cull appealed to others to collaborate now that improved methodology in neuropsychometric assessment is available.

The second paper addressed the measurement of psychosocial morbidity and was given by Dr Stirling Moorey from the CRC Psychological Medicine Group at the Royal Marsden Hospital. It was suggested that many cancer patients have a mixture of depressive and anxiety symptoms and that it would be useful to measure a general level of distress rather than separate dimensions of depression and anxiety. The Royal Marsden Group, therefore, tested the Hospital Anxiety and Depression Scale (Zigmond & Snaith, 1983) as a 14 item scale, to find out whether its reliability was improved against using the two subscales each of seven items. A heterogenous sample of 575 cancer patients, with predominantly primary disease, completed the HADS; 9% were probable cases of depression and 27% probable cases of anxiety, using a cut off score of 8 for each subscale. The mean depression score was 3.0 and mean anxiety score 5.4. A factor analysis was carried out on the complete HADS. On principal components analysis using both orthogonal and oblique rotations two factors emerged accounting for 53% of the variance which corresponded to depression and anxiety. One item on the anxiety subscale of the HADS loaded higher on the depression factor ('I can sit at ease and feel relaxed') and this was considered due to the different wording of this question compared with the other six anxiety items. All the items on the depression subscale loaded higher for depression than anxiety. Dr Moorey concluded that the HADS was measuring two constructs, depression and anxiety. The responses of females (71% sample) were compared with males (29%). The factor structure results were again upheld, except for two depression items ('feel slowed down', 'lost interest in appearance') which loaded just below the cut off of 0.45 in the female sample. The study therefore confirmed the existence of two stable factors and found that their internal consistency was high (Cronbach's alpha for anxiety 0.93, depression 0.90). Dr Moorey concluded that the HADS should continue to be applied using the two subscales.

The third session 'Can we learn anything of value from the Bristol Centre' was the subject of debate between Professors Baum (Royal Marsden Hospital, London) and Sikora (Hammersmith Hospital, London).

Professor Baum opened the debate by suggesting that patients with cancer need cure, prolongation of life and enhancement of quality of life. To achieve these objectives a partnership is needed between medical science, complementary health care and faith. Medical science cannot only help patients to get better but to feel better, and complementary care may make patients feel better and also get better and spiritual solace can help patients live and feel better. Professor Baum believed that the title of the debate should be reversed to: 'Does the Bristol Cancer Help Centre have anything to learn from us', as he believes they do.

Professor Baum first considered length of life in patients with breast cancer. A recent world overview has shown that the appropriate use of adjuvant systemic therapy can reduce the hazard of death over a 10 year period by over 25%. Professor Baum noted that although we all agree that in many ways the recent Lancet study (Bagenal *et al.*, 1990) was flawed, even the most generous interpretation would not suggest that the Bristol regimen prolongs life.

Next, Professor Baum considered quality of life. Local control of breast cancer is vital for quality of life. He cited examples of patients in extreme distress who had been treated with complementary therapy alone and had severe, fungating cancers on their chest. It is not sufficient just to aim to improve quality of life; systematic evaluation using clear outcome measures of quality of life and identified interventions is needed.

Professor Baum suggested four reasons why patients appear to vote with their feet and attend the Bristol Centre. First, unrealistic expectations, second to get away from 'bad doctors', third patients with a personality trait who want to maintain a locus of control and finally, confusion, where patients feel the need for spiritual support but cannot find this in their society. The way out of this morass is through science and working with the Bristol Centre and investigating their claims of enhancing quality of life. He warned of the danger of faith masquerading as science because this results in the uncontrolled fungating cancer on the chest wall which has been treated by wishful thinking.

Responding in favour of alternative therapy and the Bristol Centre, Professor Sikora looked at four situations: early breast cancer, early prostate cancer, inoperable lung cancer and metastatic colorectal cancer. There are many different ways that science has resulted in dealing with these diseases: for example, the type of operation given for breast cancer varies from consultant to consultant; and men with early prostate cancer seeing a urologist will be given a prostatectomy, whereas men seeing a radiotherapist will be given radiotherapy.

The Bristol Centre is about bringing the two side of orthodox and alternative (or complementary) therapy together; extremists are not helpful to patients as patients need a balanced point of view. Professor Sikora believed that the Bristol Centre has given us that balance.

Surveys by Professor Sikora and Dr Slevin have shown that many patients are already using alternative therapies. Professor Sikora has found that of patients attending the Hammersmith Hospital, London, 10% were using some form of complementary therapy for cancer and 33% were using complementary therapy for other conditions, such as arthritis.

Professor Sikora summarised the values of the Bristol Centre as:

(i) identifying unrecognised needs in cancer patients;
(ii) indicating that patients want to help themselves and it is our responsibility to focus their activity;
(iii) the Bristol Centre has shown us not to use the rigid medical model.

The discussion which followed covered the publishing of the evaluation of the Bristol Centre, whether a randomised design would have been possible for the evaluation of individual therapies such as the Bristol diet, the importance of considering the needs of individual people who have cancer and whether the Bristol Centre has contributed anything above the work carried out for many years in the United States of America.

In his closing remarks Professor Baum reminded the audience of the success of the medical model in the last 100 years in surgery, conquering infectious disease, diabetes, tuberculosis, renal disease etc. Professor Baum believes that alternative treatments for cancer will become less popular once we find a way for improving the medical model.

In his closing remarks, Professor Sikora stated that he disagreed with Professor Baum's views about the Bristol Centre and concluded that we need to look at the Bristol Centre and find out which parts of it are important and why some patients find it helpful and others do not, and then bring these back into orthodox medicine. This is what his project at the Hammersmith Hospital aims to do, and the cost of their project to date has been minuscule compared to the cost of a cancer centre.

### Communication and intervention

There were three papers in this final session, two of which addressed the issue of communication skills in cancer care, and the third described the psychotherapy trial currently being undertaken at the Royal Marsden Hospital.

Mrs Susie Wilkinson (Stockport Health Authority) reported a study investigating the extent to which ward nurses had difficulty in communicating with patients when taking a nursing history on hospital admission. She sought to clarify whether the nurses lacked communication skills or had skills which they were not using. The aim of the study was to identify those factors predictive of a communication style which facilitated discussion of patient's problems with coverage of emotional as well as physicial topics. Six randomly selected wards within a cancer hospital and a District General Hospital were sampled. Fifty-four registered and enrolled nurses were audiotaped during three types of interview: (i) a newly diagnosed patient; (ii) a patient with recurrent disease; and (iii) a patient admitted for palliative care. In general the level of facilitative communication was poor, with communication between patients admitted with recurrent disease and nurses causing most difficulty. They also completed three self-assessment scales: Speilberger *et al.*'s state Trait Anxiety Inventory (1983), Collett and Lester's Fear of Death Scale (1969) and Norbeck *et al.*'s Social Support Questionnaire (1981).

The environment created by the ward Sister was the single most important predictor of facilitative communication reflecting the ward Sister's own communication skills as a role model. Interestingly, having attended an oncology course was associated with facilitative communication whilst having attended a communication skills course was not. It was suggested that the knowledge and attitudes gained from an oncology course provide an important background to acquiring necessary communication skills.

Nurses own religious beliefs e.g. atheism, and fear of dying, predicted blocking of communication about emotionally loaded topics. Nurses who blocked communication were less anxious after patient interviews than those who had facilitated communication about difficult topics. The predictors identified by Mrs Wilkinson are potentially a useful aid to recruitment and eduction of nurses.

In the second part of this session, Dr Maura Hunt (Regional Nursing Research Officer, South East Thames Regional Health Authority) drew attention to the advocacy in nursing literature of informality of approach and reported a qualitative analysis of audiotaped conversations between five symptom control nurses and 54 of their patients visited at home.

Four concepts of role formats were identified: bureaucratic (e.g. use of collegial authority, compilation of records), biomedical (e.g. taking of illness history), social therapy (e.g. talking about cancer, expressing feelings) and friendly/informal. Dr Hunt described the characteristics of the latter as including the use of first names, no wearing of uniform, social talk, self disclosure and generally unlike hospital services. She then reported on the extent to which these characteristics appeared in the nurses interactions with their patients.

They overtly professed an informal friendly approach in introducing themselves to patients and their carers and subsequently used this role format to fill in the time while waiting to see the patient, as a boundary between activities and as a preclosure sequence to signal the imminent end of the visit. While the use of first names, lack of uniform and emphasis on difference from hospital were easily identified, self disclosure (e.g. the extent to which staff shared information with patient) was little sought or taken up by patients. Dr Hunt pointed out the difference between service giving and social interaction emphasising the need to maintain some formality in order to meet the objectives of the service.

The discussion which followed this talk took up this point with questions about whether neutral conversation represented time which might have been better spent covering relevant issues. The paper concluded that whilst some informality may be seen as desirable, there is a need for it to be kept in check in order for service goals to be accomplished.

The final paper of the day was presented by Dr Maggie Watson (CRC Psychological Medicine Group, Royal Marsden Hospital) who reviewed the literature linking psychological responses of cancer patients to disease outcome. She sought to bring together two parallel strands of research concerning emotional control and fighting spirit, both of which have been related to cancer prognosis, and cautioned against premature claims made on the basis of the fragile data currently available.

Dr Watson presented an update on the progress of the trial of Adjuvant Psychological Therapy conducted at the Royal Marsden Hospital. A consecutive series of 1200 patients has now been screened for psychological distress and the 23% of the sample who scored high for psychological morbidity were invited to take part in the adjuvant psychotherapy trial. The aim of this intervention is to maximise patients' involvement in rewarding aspects of life unrelated to cancer and to enable more constructive use of the time spent focusing on the disease, for example in appropriate expression of emotion.

Dr Watson proposed a model of the cycle by which psychological morbidity may develop. The person with a type C personality and a fatalistic attitude, is hypothesised to respond to a diagnosis of cancer by blocking emotional expression, controlling anger and anxiety and thereby experiencing feelings of hopelessness and helplessness. These are then associated with increased psychological morbidity i.e. higher levels of anxiety and depression. The discussion following this paper pursued the issue of whether these psychological responses could be changed, and how change could occur through psychotherapy. Dr Watson pointed out the tentative nature of the model and emphasised the need for continuing research.

The conference was closed by the Chairman of the BPOG, Dr Maurice Slevin.

The eighth annual conference will be held in London on December 9–10 1991. Anyone wishing to join the BPOG should contact the Secretary, Dr Irene Higginson, Department of Community Medicine, University College and Middlesex School of Medicine, 66–72 Gower Street, London WC1E 6EA.

**References**

BAGENAL, F., EASTON, D., HARRIS, E., CHILVERS, C. & MC-ELWAIN, T. (1990). Survival of patient with breast cancer attending the Bristol Cancer Help Centre. *Lancet*, **336**, 606.

COLLETT, J. & LESTER, D. (1969). The fear of death and the fear of dying. *J. Psychol.*, **72**, 179.

GRAY, R.E. & DOAN, B.D. (1990). Heroic self-healing and cancer: clinical issues for the health profession. *J. Palliative Care*, **6**, 32.

NELSON, H.E. (1982). *National Adult Reading Test (NART)*: Test Manual, Windsor, NFER-Nelson.

NORBECK, J.S., LINDSEY, A.M. & CARRIERI, Z.L. (1981). The development of an instrument to measure social support. *Nursing Res.*, **30**, 264.

SPEILBERGER, C.D., GORSCH, R.L., LUSHENE, R.E., VAGG, P.R. & JACOBS, G.A. (1983). *Manual for the State-Trait Anxiety Inventory*, Palo Alto, California Counselling Psychologists Press.

ZIGMOND, A. & SNAITH, R. (1983). The hospital anxiety and depression scale. *Act. Psychiat. Scand.*, **67**, 361.